# *Ex vivo* mass spectrometry-based biodistribution analysis of an antibody-Resiquimod conjugate bearing a protease-cleavable and acid-labile linker

**DOI:** 10.3389/fphar.2023.1320524

**Published:** 2023-12-06

**Authors:** Lydia Bisbal Lopez, Domenico Ravazza, Matilde Bocci, Aureliano Zana, Lucrezia Principi, Sheila Dakhel Plaza, Andrea Galbiati, Ettore Gilardoni, Jörg Scheuermann, Dario Neri, Luca Pignataro, Cesare Gennari, Samuele Cazzamalli, Alberto Dal Corso

**Affiliations:** ^1^ Chemistry Department, Università degli Studi di Milano, Milano, Italy; ^2^ R&D Department, Philochem AG, Otelfingen, Switzerland; ^3^ Department of Chemistry and Applied Biosciences, Swiss Federal Institute of Technology (ETH Zürich), Zürich, Switzerland; ^4^ Philogen S.p.A, Siena, Italy

**Keywords:** immune stimulating antibody conjugates, Resiquimod, toll-like receptors, prodrugs, quantitative biodistribution

## Abstract

Immune-stimulating antibody conjugates (ISACs) equipped with imidazoquinoline (IMD) payloads can stimulate endogenous immune cells to kill cancer cells, ultimately inducing long-lasting anticancer effects. A novel ISAC was designed, featuring the IMD Resiquimod (R848), a tumor-targeting antibody specific for Carbonic Anhydrase IX (CAIX) and the protease-cleavable Val-Cit-PABC linker. *In vitro* stability analysis showed not only R848 release in the presence of the protease Cathepsin B but also under acidic conditions. The *ex vivo* mass spectrometry-based biodistribution data confirmed the low stability of the linker-drug connection while highlighting the selective accumulation of the IgG in tumors and its long circulatory half-life.

## 1 Introduction

The conjugation of small molecule drugs to tumor-targeting antibodies is a well-established strategy to increase the accumulation of these compounds at the tumor site, limiting their toxicity to healthy organs. Fourteen antibody-drug conjugates (ADCs) have now reached the market, while more than a hundred ADC prototypes are currently at various stages of clinical evaluation (Maiti et al., 2023).

Within the modular structure of ADCs, the payload is arguably the direct mediator of both therapeutic effects and side toxicity. Highly potent cytotoxic agents of different classes are currently used as payloads in the vast majority of ADCs (Conilh et al., 2023). Cytotoxic drugs kill cells in rapid proliferation by interfering with fundamental processes of the cell life cycle (e.g., mitosis and DNA replication). Due to the high heterogeneity of tumor cell populations, the active delivery of these agents typically leads to a partial eradication of neoplastic lesions and to the development of drug-resistant cancer cells whose pharmacological treatment is more cumbersome (Yu et al., 2013; Yu et al., 2015; Takegawa et al., 2017).

In the search for more efficacious and durable therapies, the involvement of immune cells and the stimulation of immune-mediated anticancer responses have become a standard in oncology. In the ADC context, this approach has been pursued by the selection of cytotoxic agents capable of inducing immunogenic cell death and by the ADC combination with new-generation therapeutics, such as immune-checkpoint inhibitors (D’Amico et al., 2019; Rios-Doria et al., 2017; Gerber et al., 2016; Cazzamalli et al., 2018a).

**FIGURE 1:**
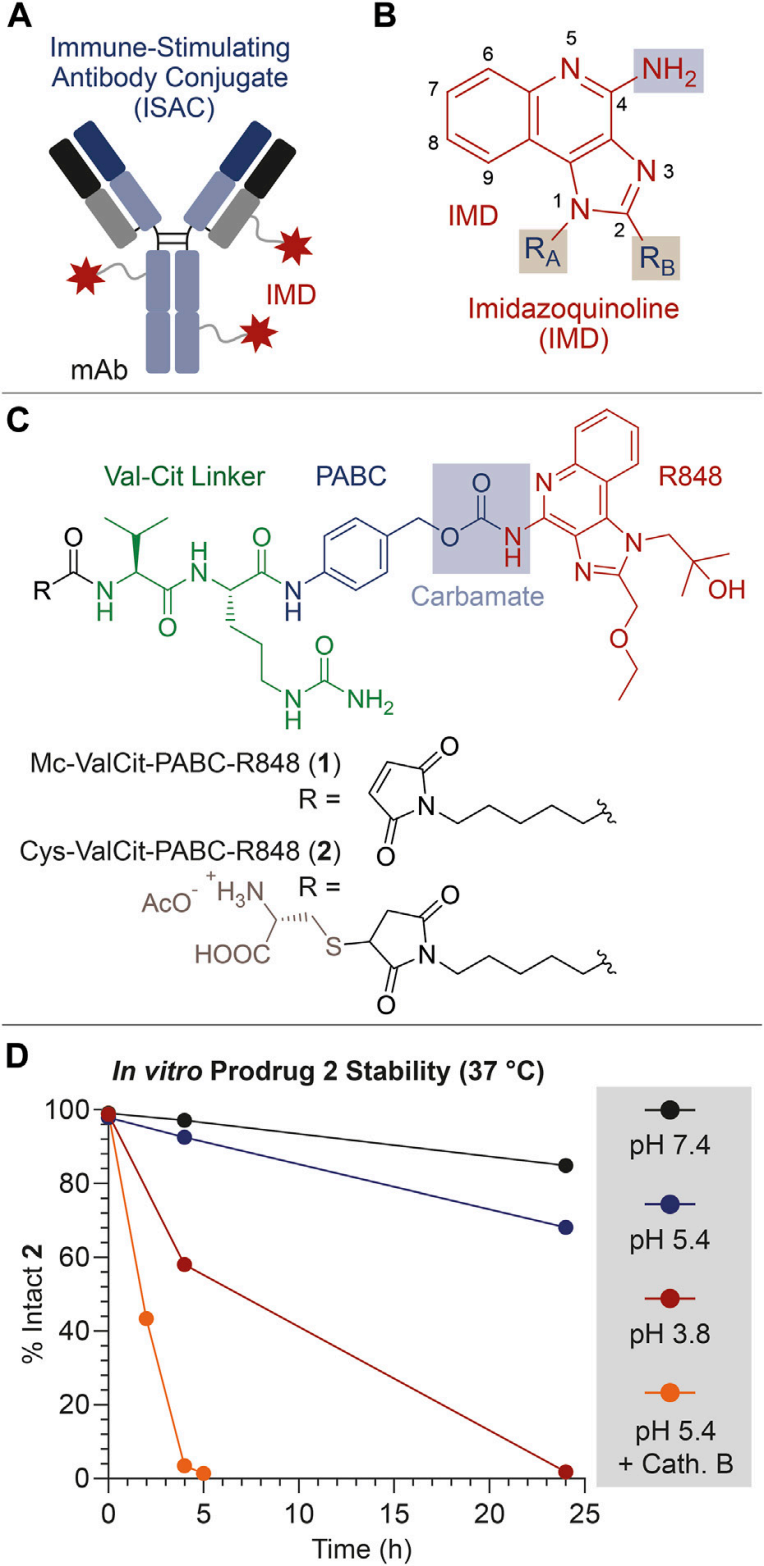
**(A)** Schematic representation of an ISAC product. **(B)** General molecular structure of IMDs typically used as TLR7/8 agonists. **(C)** Molecular structure of the linker-drug module **1**, bearing the Val-Cit linker (green), the PABC self-immolative spacer (blue) and the IMD payload Resiquimod (**R848**, red). The structure of the derivative **2** is also shown, consisting in a cysteine-thioether derivative of **1**. **(D)** *In vitro* stability of **2** under different conditions (neutral/acidic pH, and presence/absence of the protease Cathepsin B). All drawings, structures and graphics depicted in Panel 1 and throughout the present article have been edited by the authors.

More recently, the use of immunomodulating small molecules as ADC payloads has been proposed as an alternative to traditional cytotoxic agents (Ackerman et al., 2021; Fang et al., 2022; Brant et al., 2023). Immune-stimulating antibody conjugates (ISACs, Figure 1A) are based on tumor-targeting monoclonal antibodies (mAbs) functionalized with small molecule agonists of Toll-Like Receptors 7 and 8 (TLR7/8) such as imidazoquinoline derivatives (IMDs) (Bhagchandani et al., 2021). Here, IMDs are typically derivatized with uncleavable linkers connected at the imidazole N-1 atom (Figure 1B) (Wang et al., 2020; Bolli et al., 2021; Huppertsberg et al., 2021; Slezak et al., 2022). This design exposes the free aniline group at position 4, which represents a fundamental pharmacophoric portion of this class of molecules (Talukdar et al., 2021). As a result, ISACs can exhibit significant immune-reactivity also in their intact form. This may pose the risk of systemic toxicity, resulting from the uncontrolled activation of leukocytes in the blood and secondary lymphoid organs. Conceivably, this risk is further amplified by the intrinsic immunogenicity of ADCs and by their long circulatory half-lives (Blaich et al., 2016).

In a different design of linker-drug modules, the IMD aniline group is connected to the protease-cleavable Valine-Citrulline (Val-Cit) dipeptide through the *para*-aminobenzylcarbamate (PABC) self-immolative spacer (Figure 1C). With this design, the aniline transformation into carbamate is expected to silence the IMD pharmacological activity, which can be selectively restored at the tumor site through the proteolytic cleavage of the Val-Cit linker and fast degradation of the PABC spacer. This linker-IMD module has been connected to various classes of carriers (Keen et al., 2019; Pietersz, 2019; Hingorani et al., 2021; Fang et al., 2022). In this work, we describe the use of this cleavable and supposedly safe linker-spacer module to connect the aniline moiety of the well-known IMD Resiquimod (R848) to a mAb, specific for the tumor receptor Carbonic Anhydrase 9 (CAIX).

## 2 Materials and methods

### 2.1 Chemical synthesis

#### 2.1.1 *Synthesis of Mc-ValCit-PABC-R848* **1**

Mc-ValCit-PABC-PNP (PNP: *para*-nitrophenol carbonate; MedChemExpress, 48 mg, 66 μmol, 2.2 equiv.) was dissolved in DMF (955 μL). R848 (9 mg, 30 μmol, 1 equiv.), HOAt (1 mg, 6 μmol, 0.2 equiv.) and *i*Pr_2_NEt (15 μL, 84 μmol, 2.8 equiv.) were added. The mixture was stirred for 48 h at room temperature. The solution was concentrated *in vacuo* and the product was purified by reverse-phase HPLC (20% MeCN to 70% over 15 min, *t*_R_: 9.5 min). The collected fractions were concentrated *in vacuo* and lyophilized. The resulting powder was purified again by HPLC (gradient from 30% to 55% MeCN in H_2_O + 0.1% trifluoroacetic acid (TFA) over 8 min, *t*_R_: 6.8 min) and the collected fractions were lyophilized to give Mc-ValCit-PABC-R848 **1** as a white solid (7 mg, 7.5 μmol, 59%).

#### 2.1.2 *Synthesis of Cys-ValCit-PABC-R848* **2**

Mc-ValCit-PABC-R848 **1** (7 mg, 7.5 μmol, 1 equiv.) was dissolved in DMF (1.2 mL). A solution of L-Cys (1 mg, 5.8 μmol, 1 equiv.) in PBS (1.2 mL) was added. NaHCO_3_ (120 μL) was added and the mixture was stirred for 2 h at room temperature. The solution was concentrated *in vacuo* and the crude product was purified by HPLC (20% MeCN to 55% over 7 min, *t*_R_: 4.4 min). The collected fractions were concentrated *in vacuo* and lyophilized. The resulting powder was purified again by HPLC (gradient from 20% to 45% MeCN in H_2_O + 0.1% TFA over 6 min, *t*_R_: 4.5 min) and the collected fractions were lyophilized to give Cys-ValCit-PABC-R848 **2** as a white solid (3 mg, 3.2 μmol, 42%).

Further details on materials and compound characterizations are included in the Supplementary Material.

### 2.2 *In vitro* stability assays

#### 2.2.1 Stability under neutral and acidic conditions

A solution of Cys-ValCit-PABC-R848 **2** (10 μL, 20 mM solution in DMSO) was added to 200 μL of aqueous buffer (acetate buffer pH 3.8 or 5.4; phosphate buffer pH 7.4). The mixture was incubated at 37°C. Aliquots (50 μL) were taken at different timepoints, diluted in an 8:2 H_2_O:MeCN solution (350 μL) and analyzed by HPLC.

#### 2.2.2 Cathepsin B cleavage assay

The enzymatic stability assay was performed following a published procedure (Cazzamalli et al., 2016). Briefly, Cathepsin B from human placenta (Merck, cat. code C0150) was dissolved in water (0.4 mg in 400 μL) and a 50-μL aliquot was diluted in acetate buffer pH 5.4 (50 μL). 100 μL of a 1:1 (*v/v*) 30 mM DTT:15 mM EDTA was added. The mixture was incubated at room temperature for 15 min. A solution of **2** (10 μL, 20 mM solution in DMSO) was added. The mixture was incubated at 37°C. Aliquots (50 μL) were taken at different timepoints and diluted in MeCN + 0.1% TFA (300 μL). The diluted aliquots were centrifuged at 14,500 rpm for 10 min and the supernatant was concentrated *in vacuo*. The remaining solid was then suspended in an 8:2 H_2_O:MeCN mixture (350 μL) and analyzed by HPLC.

#### 2.2.3 HPLC analysis

The samples were injected in into an analytical HPLC-PDA system (see the Supplementary Material). H_2_O + 0.1% TFA (solvent A) and MeCN + 0.1% TFA (solvent B) were used as the mobile phase at a flow rate of 1 mL/min. The gradient was programmed from 5% to 30% B over 26 min. Areas under the curve (AUC) of the detected peaks were measured using the Empower software. The rate of R848 release from the starting carbamate **2** was obtained by calculating the relative ratios of AUC values corresponding to the prodrug **2** and the free payload. Data were plotted versus time using GraphPad Prism software.

### 2.3 ISAC **3** preparation

**FIGURE 2:**
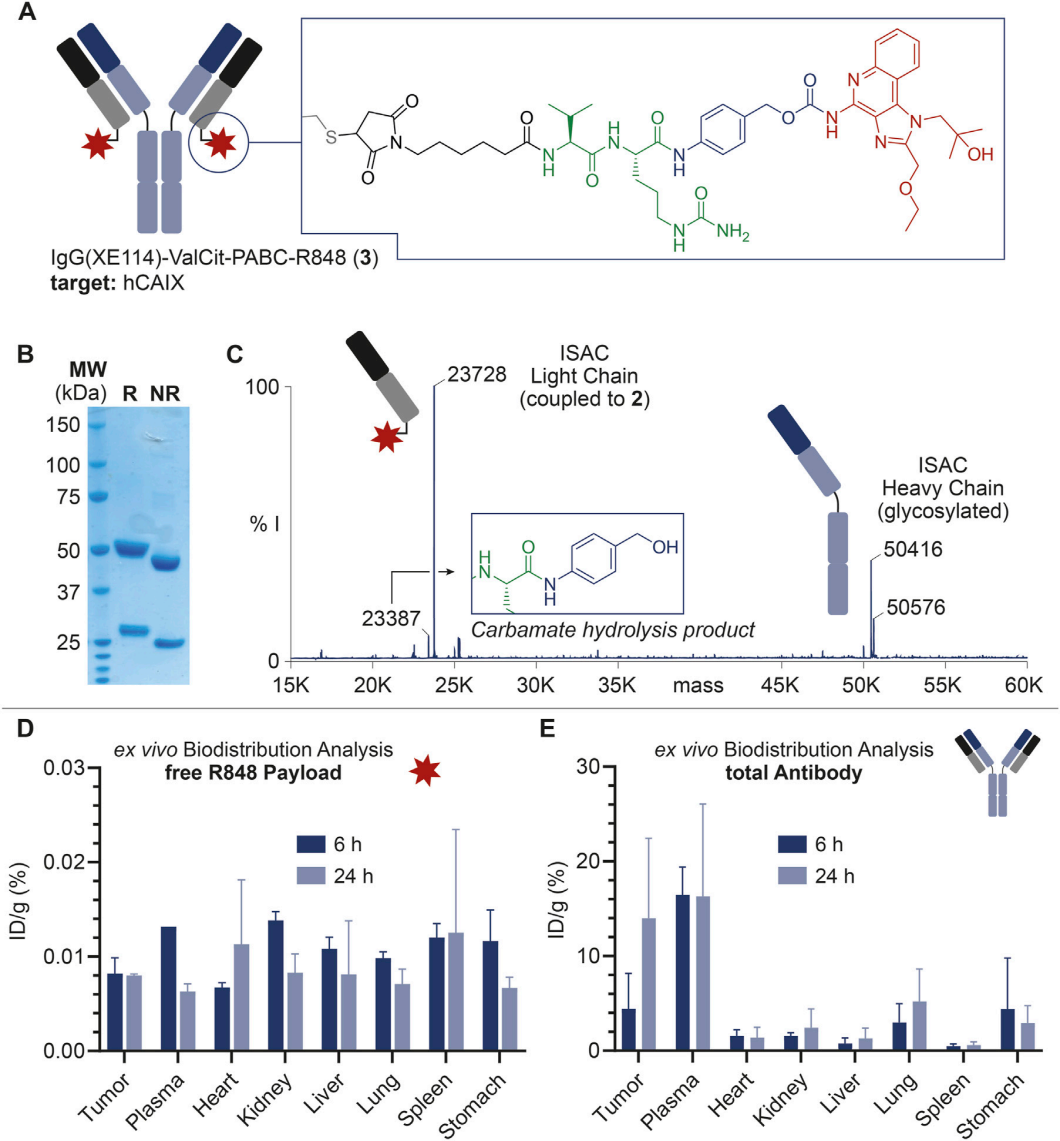
**(A)** Schematic representation of ISAC **3**, including the IgG structure and site of conjugation of the linker-drug module. ISAC **3** features IgG(XE114) as the mAb carrier, R848 as the payload, and the Val-Cit-PABC module as the linker. The linker-drug module is connected to the mAb at the C-terminal Cys residues in the mAb light chains (DAR = 2). **(B)** SDS-PAGE analysis of **3**. R: reducing conditions; NR: non-reducing conditions; MW: molecular weight. **(C)** Deconvoluted ESI-MS spectrum of **3** and assignment of main peaks. In addition to the MS peaks relative to the ISAC heavy chain and payload-bearing light chain, a low-intensity peak corresponding to the carbamate hydrolysis and loss of payload is also detectable, with a 340-Da lower mass than the intact bioconjugate. % I: = % of MS intensity. **(D)** *Ex vivo* MS-based biodistributions of R848 released from **3**, 6 and 24 h post-injection. **(E)** *Ex vivo* MS-based biodistributions of the total antibody, 6 and 24 h post-injection.

The antibody IgG1(XE114) was produced and purified according to a published procedure (Cazzammalli et al., 2018b). IgG1(XE114) was incubated with TCEP·HCl (60 equiv., i.e. 30 equiv. per reactive cysteine residue) in PBS (pH 7.4) overnight at room temperature. The protein was then concentrated to approx. 1 mg/mL. A solution of **1** (20 equiv., i.e. 10 equiv. per reactive cysteine residue) in DMSO was added to the protein (10% *v/v* final DMSO concentration), which was stirred for 2 h at room temperature. The mixture was then purified by PD10 desalting column (Cytiva). OD at an absorbance of 280 nm (OD_280_) was measured and protein-containing fractions (OD_280_ > 0.1 mg/mL) were pooled. ISAC **3** featured a drug/antibody ratio (DAR) of 2, resulting from the site-specific payload conjugation to the C-terminal Cys residues at the mAb light chain. ISAC **3** characterization is reported in Figure 2 and in the Supplementary Material.

### 2.4 Ethics approval

Animal experiments were conducted in accordance with Swiss animal welfare laws and regulations under the license number ZH006/2021 (ethic approval) granted by the Veterinäramt des Kantons Zurich. This study was designed and performed in accordance with the ARRIVE guidelines.

Since the complexity of living organisms cannot be adequately reproduced by *in vitro* laboratory procedures (e.g., cells cultured in a petri dish), the use of tumor-bearing mice was necessary to evaluate the tumor targeting and the drug release properties of antibody conjugates presented in this article.

*In vivo* studies were conducted on tumor-bearing female Balb/c nude mice (Janvier, 6–8 weeks old). Mice were kept in a specific pathogen-free (SPF) laboratory, randomly assigned to different treatment groups. Animals were housed in cages (maximum of 5 mice per cage) equipped with fine bedding, environmental enrichment (i.e., polycarbonate cottage and nestlets), and *ab libitum* food and water. Animal wellbeing was checked on a regular basis (daily, during the week; during the weekend if abnormal behavior or health-status was observed).

The procedures reported in this work did not require prior administration of anesthetic drugs since they produced only mild and brief discomfort to the study animals (severity degree = 1). During the study, mice were checked daily for signs of pain and discomfort.

### 2.5 Implantation of subcutaneous tumors

Upon thawing, CT26.3E10 tumor cells were maintained in DMEM medium (Gibco) supplemented with fetal bovine serum (10%, FBS, Gibco) and Antibiotic-Antimycotic (1%, AA, Gibco) at 37°C and 5% CO_2_ (Cazzamalli et al., 2018a). For passaging, cells were detached using Trypsin-EDTA 0.05% (Gibco) when reaching 80%–90% confluence. CT26.3E10 cells were grown to 80% confluence and detached with Trypsin-EDTA 0.05% (Gibco). Cells were re-suspended in Hanks′ Balanced Salt solution (HBSS, Gibco) to a final concentration of 5 × 10^7^ cells/mL. Aliquots of 5 × 10^6^ cells (100 μL of the suspension) were injected subcutaneously in the right flank of female immunocompetent BALB/c mice (Janvier, 6–8 weeks old), without previous anesthesia.

### 2.6 Biodistribution studies

CT26.3E10 tumors were implanted as described above and allowed to grow to an average volume of ca. 200 mm^3^. Tumor-bearing mice (*n* = 2 per timepoint) were injected intravenously with test compounds (2.5 nmol/mouse) and sacrificed at 6 h and 24 h post injection (total of 4 animals). Mice were euthanized by CO_2_ asphyxiation (flow = 12 cm^3^/min), without prior anesthesia. Few minutes after the animal stopped breathing, fresh blood was withdrawn by hearth puncture with a needle syringe and collected in lithium heparin tubes (BD Microcontainer LH Tubes), vortexed and centrifuged (15,000 X G, 15 min). Collected plasma was frozen and stored at −80 °C. Healthy organs and tumors were subsequently excised, frozen with dry ice and stored at −80°C.

### 2.7 mAb quantification by LC-MS analysis

#### 2.7.1 Sample preparation

Samples (40 mg) were resuspended in 800 μL of lysis buffer (0.25% sodium deoxycholate, 1 mM EDTA, 0.5% Igepal in PBS pH 7.4, and protease inhibitor (Roche)), the internal standard was added and the samples were homogenized with a tissue lyser (TissueLyser II, QIAGEN) for 30 min at 30 Hz. After homogenization, lysates were put on rotation at 4°C for 2 h, centrifuged at 21,000 *g* for 30 min. The supernatants were recovered and incubated with previously blocked protein A magnetic beads (Merck) for 2 h in rotation at 4°C. Beads were recovered and washed with lysis buffer, buffer A (150 mM NaCl, 20 mM Tris in water, pH 7.5) and buffer B (400 mM NaCl, 20 mM Tris in water, pH 7.5). Proteins were eluted with glycine (0.1 M, pH 3) and incubated shaking for 30 min. Cysteines were reduced and carbamylated with TCEP·HCl and IAA respectively. Proteins were finally digested overnight with trypsin at 37°C. Tryptic peptides were purified on C_18_ Macrospin columns (Harvard Apparatus) according to the manufacturer’s instructions and eluates were dried at room temperature with a vacuum centrifuge (Eppendorf). Dried samples were finally resuspended in a solution of 3% MeCN and 0.1% FA (25 μL). Samples (6 µL) were then injected in the nanoLC-MS system.

#### 2.7.2 nanoLC-MS analysis

Chromatographic separation was carried out on an Acclaim PepMap RSLC column (50 μm × 15 cm, particle size 2 μm, pore size 100 Å, Thermo Fisher Scientific) with a gradient program from 100% A (H_2_O + 0.1% FA), 0% B (MeCN + 0.1% FA) to 65% A, 35% B in 60 min on an Easy nanoLC 1000 (Thermo Fisher Scientific). The LC system was coupled to a Q-Exactive mass spectrometer (Thermo Fisher Scientific) via a Nano Flex ion source (Thermo Fisher Scientific). Ionization was carried out with the following settings: spray voltage 2 kV; capillary temperature 250°C; S-lens RF level 60. The mass spectrometer operated in scheduled Single Ion Monitoring (schedule-SIM-MS2) mode with the following parameters: isolation window of targeted-SIM 6 *m/z*, resolution of 70,000 (FWHM at 200 *m/z*), maximum injection time 250 ms and AGC target 2 × 105. MS/MS spectra were recorded at a resolution of 17,500 and a maximum injection time of 250 ms.

MS/MS data were searched against a FASTA file containing the mouse reference proteome, common contaminant proteins, the analyte, and the internal standard sequences. The following analysis settings were used with SEQUEST in PD2.5: digestion enzyme Trypsin; precursor mass tolerance 10 ppm; fragment mass tolerance 0.02 Da; one static modification (Carbamidomethylation of cysteine); one variable modification (oxidation of methionine). False discovery rates were calculated with Percolator.

The data was analyzed with Skyline v22.2.0.351. For the analyte and the internal standard, only unique peptides with the highest signal intensity were selected and their peak areas was integrated. Protein abundances were obtained as mean of peptides area. In each sample a ratio analyte/IS was calculated. The ratios were then transformed into pmol/g of tissue using single concentration external calibration point and corrected by the total weight of the sample analyzed. The percentage of injected dose per gram (%ID/g) was finally determined by normalizing the value based on the total dose injected into the mouse.

### 2.8 R848 quantification by LC-MS analysis

#### 2.8.1 Sample preparation

Murine frozen plasma (50 µL) and tissues (ca. 50 mg) collected from the sacrificed mice (see Section 2.5) were thawed, and 600 μL of a 95% MeCN solution containing 0.1% formic acid (FA) were added to induce protein precipitation. 50 μL of an internal standard solution (300 nM, aq. solution of R848-d_5_ purchased from MedChemExpress, containing 3% MeCN and 0.1% FA) was added to the mixture. Sample homogenization was carried out with a tissue lyser (TissueLyser II, QIAGEN) for 15 min at 30 Hz. Once homogenized, the samples were centrifuged (21,000 g for 10 min) and the supernatants were collected and dried at room temperature using a vacuum centrifuge (Eppendorf). Pellets were resuspended in an aq. solution of 3% MeCN and 0.1% TFA (1 mL) and purified on Oasis HLB SPE columns (1 mL volume, 30 mg sorbent, Waters) according to manufacturers’ instructions. Eluates were dried under vacuum at room temperature. Dry pellets were resuspended in a 3% MeCN and 0.1% TFA solution in water (400 μL) and purified on C_18_-Macro-Spin SPE columns (Harvard Bioscience). Eluates were completely dried under vacuum at room temperature, resuspended in an aqueous solution of 3% MeCN and 0.1% FA (50 μL), of which 1 µL was injected into the LC-MS system.

#### 2.8.2 LC-MS analysis

Chromatographic separation was performed on a Hypersil GOLD column (100 mm × 2.1 mm, particle size 1.9 μm, pore size 175 Å, Thermo Fisher Scientific) on a Vanquish UHPLC System (Thermo Fisher Scientific) with the following 6 min gradient program: 1) 95% A (H_2_O: FA, 99.9 : 0.1), 5% B (MeCN: FA, 99.9 : 0.1) for 0.1 min; 2) from 1% to 35% A, 65% B in 2.4 min; 3) from 2% to 5% A, 95% B in 0.4 min; 4) 5% A, 95% B for 1.3 min; 5) 95% A, 5% B for in 1.8 min. The flow rate was set at 600 μL/min, and the column was thermostatted at 50°C. The LC system was coupled to a Q-Exactive mass spectrometer (Thermo Fisher Scientific) via an Ion Max-S API Source (Thermo Fisher Scientific). Ionization was carried out with the following parameters: spray voltage 3 kV; capillary temperature 380°C; S-lens RF level 60. Mass spectrometry analysis was done in positive ion mode with the following parameters: resolution 70,000 (FWHM at 200 *m/z*); AGC target 5 × 104; maximum injection time 200 ms; isolation window 14 *m/z*; isolation offset 5 *m/z*. The detector was operating in targeted Single Ion Monitoring mode (t-SIM) following the transition 315.18155 *m/z*.

The data was analyzed with Skyline v22.2.0.351. In each sample, a ratio analyte/IS was calculated. The ratios were then transformed into pmol/g of tissue using single concentration external calibration point and corrected by the total weight of the sample analyzed. The percentage of injected dose per gram (%ID/g) was finally determined by normalizing the value based on the total dose injected into the mouse.

## 3 Results

### 3.1 Chemical synthesis

The aniline group of R848 was connected to the protease-cleavable dipeptide Val-Cit through the well-known PABC self-immolative spacer (Dal Corso et al., 2019). The resulting Mc-ValCit-PABC-R848 **1** module (Figure 1B) underwent a thiol-maleimide Michael addition with free Cysteine (Cys), leading to the model prodrug **2**. This modification was devised to increase the solubility of the linker-drug module in aqueous media, facilitating the stability analysis *in vitro*. The resulting compounds **1** and **2** were purified by HPLC and characterized by mass spectrometry (see the Supplementary Material).

### 3.2 Stability analysis of **2**

In order to investigate the stability of the linker-spacer-payload connections, compound **2** was incubated in different aqueous solutions and aliquots were collected at different timepoints, following measurement of the percentage of the intact starting material by HPLC.

To confirm that **2** was a substrate for the enzyme Cathepsin B, the prodrug was treated with a catalytic amount (0.2%) of protease, following a protocol reported in the literature (Cazzamalli et al., 2016). As expected, the enzyme efficiently triggered the release of R848, and only traces of intact **2** were detected after 5 h (Figure 1D). Since the Cathepsin B cleavage assay was performed under acidic conditions, which resemble the lysosomal environment and are necessary for the enzyme activation, the stability of **2** was also evaluated in acetate buffer (pH 5.4) at 37°C in the absence of the enzyme. The incubation of **2** under these conditions led to the detection of 93% and 68% intact prodrug after 4 and 24 h, respectively. In this experiment, a +16 Da increase of the MS peaks relative to the Cys adducts were attributed to the thioether oxidation to sulfoxide (see the Supplementary Material) (Boyatzis et al., 2017). Interestingly, the prodrug stability was found to further decrease when the pH was lowered to 3.8. In this case, 58% of intact prodrug was detected after a 4-hour incubation, while the analysis at 24 h revealed only traces of **2**. In all cases, free R848 was detected as the main degradation product.

Finally, the prodrug stability under physiological conditions (phosphate buffer, pH 7.4) was also analyzed, which indicated that **2** was substantially intact (97%) after 4 h, while a minimal degradation (85%) and R848 release was observed after 24 h.

This stability analysis clearly indicates that the ValCit-PABC-R848 module can rapidly liberate the R848 payload in the presence of Cathepsin B but, unexpectedly, a slow payload release is also possible under mildly acidic conditions. The acid hydrolysis of benzyl-carbamates proceeds through the formation of a benzyl carbocation and it typically requires high concentrations of strong acids (HCl, HBr or trifluoroacetic acid) and high temperatures, such as for Cbz-protected anilines (Beveridge et al., 2020).

### 3.3 Design and preparation of ISAC **3**

The fully human IgG(XE114) mAb was used as the targeting unit for the ISAC **3**. This mAb binds the tumor-associated antigen Carbonic Anhydrase 9 (CAIX), a transmembrane enzyme expressed by Renal Cell Carcinoma (RCC) and hypoxic tumor cells, while rarely found in healthy organs (Thiry et al., 2006; Genega et al., 2010; Santos Pacheco de Campos et al., 2022). IgG(XE114) has been conjugated to the cytotoxic payload monomethylauristatin E (MMAE) and the resulting ADC showed a strong anticancer activity in mouse models (Cazzamalli et al., 2018a).

The mAb was expressed in mammalian cells, purified by affinity chromatography and characterized by SDS-PAGE and size exclusion chromatography. From the structural point of view, the mutation of three Cys residues in the heavy chain of IgG(XE114) into serine residues was devised to deplete interchain disulfide bonds, leaving each of the C-terminal Cys units in the light chains as the unique nucleophiles. With this design, following the mAb incubation with **1**, the resulting ISAC **3** exhibited a drug/antibody ratio (DAR) of 2, as confirmed by mass spectrometry analysis (Figure 2C).

### 3.4 *Ex vivo* biodistribution analysis

In order to investigate the ability of the novel CAIX-targeting ISAC **3** to accumulate in tumors and deliver the immunomodulating R848 payload, *ex vivo* mass spectrometry-based biodistribution studies in tumor-bearing mice were performed, following a similar protocol reported for MMAE conjugates (Zana et al., 2022) and specifically aimed at i) the detection of the free, released R848 and ii) total antibody quantification, regardless of its chemical functionalization with the linker-drug module. Immunocompetent Balb/c mice bearing subcutaneous CT26.3E10 xenografts (i.e., an orthotopic murine colorectal carcinoma cell line stably transfected with human CAIX) were intravenously injected with 125 nmol/kg of **3**, followed by their sacrifice after 6 and 24 h and harvesting of tumor and healthy organs. The tissues were then homogenized and processed as described in the Materials and Methods section. Finally, samples were subjected to LC-MS analysis. Commercially available deuterated R848 (R848-d_5_) was used as the internal standard to perform the free payload quantification, whereas a mAb with a different light chain isotype (approx. 42% sequence homology) was selected as the internal standard for total antibody quantification.

As shown in Figure 2D, a detectable amount of free R848 was observed in all organs and a time-dependent decrease of %ID/g values was outlined in most of the analyzed tissues. However, %ID/g values were generally low, and a preferential accumulation of the free payload in specific tissues did not emerge.

On the other hand, total mAb quantification (Figure 2E) revealed a significant tumor accumulation of IgG(XE114) at 6 h post-injection (7% ID/g), which was even more striking after 24 h (14% ID/g). Tumor-to-organ ratios higher than 3 at these timepoints were observed for all organs, except plasma.

## 4 Discussion

A novel ISAC **3**, in which the Mc-ValCit-PABC-R848 module **1** was covalently connected to a fully human antibody clone specific for the transmembrane protein CAIX was designed and isolated. CAIX represents a favorable tumor-associated antigen for ISACs due to its expression on tumor cell membranes. CAIX-specific ISACs can act as “molecular bridges” between cancer and immune cells, instructing the latter to induce an efficient therapeutic activity. In addition, the binding to antigens with low internalization rates (such as CAIX) conceivably prolongs the ISAC exposure to tumor-infiltrating immune cells (Cazzamalli et al., 2018b).

Our stability data (Figure 1D) indicates that the enzymatic digestion of Val-Cit-PABC-R848 leads to a rapid release of the R848 payload, which can be mediated *in vivo* by Cathepsin B and other tumor-associated proteases for which the Val-Cit linker is a substrate (Miller et al., 2021). However, a low stability of the PABC-R848 carbamate bond was observed in the absence of proteases. Free R848 was detected after incubation of the model construct **2** at pH 7.4 at 37°C, which warned us to avoid the prolonged handling of ISAC **3** solutions at room temperature. On the other hand, a significantly lower stability of the spacer-drug module was detected under acidic conditions. LC-MS data confirmed that the loss of free R848 in the absence of Cathepsin B occurred alongside the conversion of the PABC spacer into the corresponding benzyl alcohol (see the Supplementary Material). The latter was also detected in small amounts during the ESI-MS analysis of ISAC **3** (Figure 2C).

**FIGURE 3:**
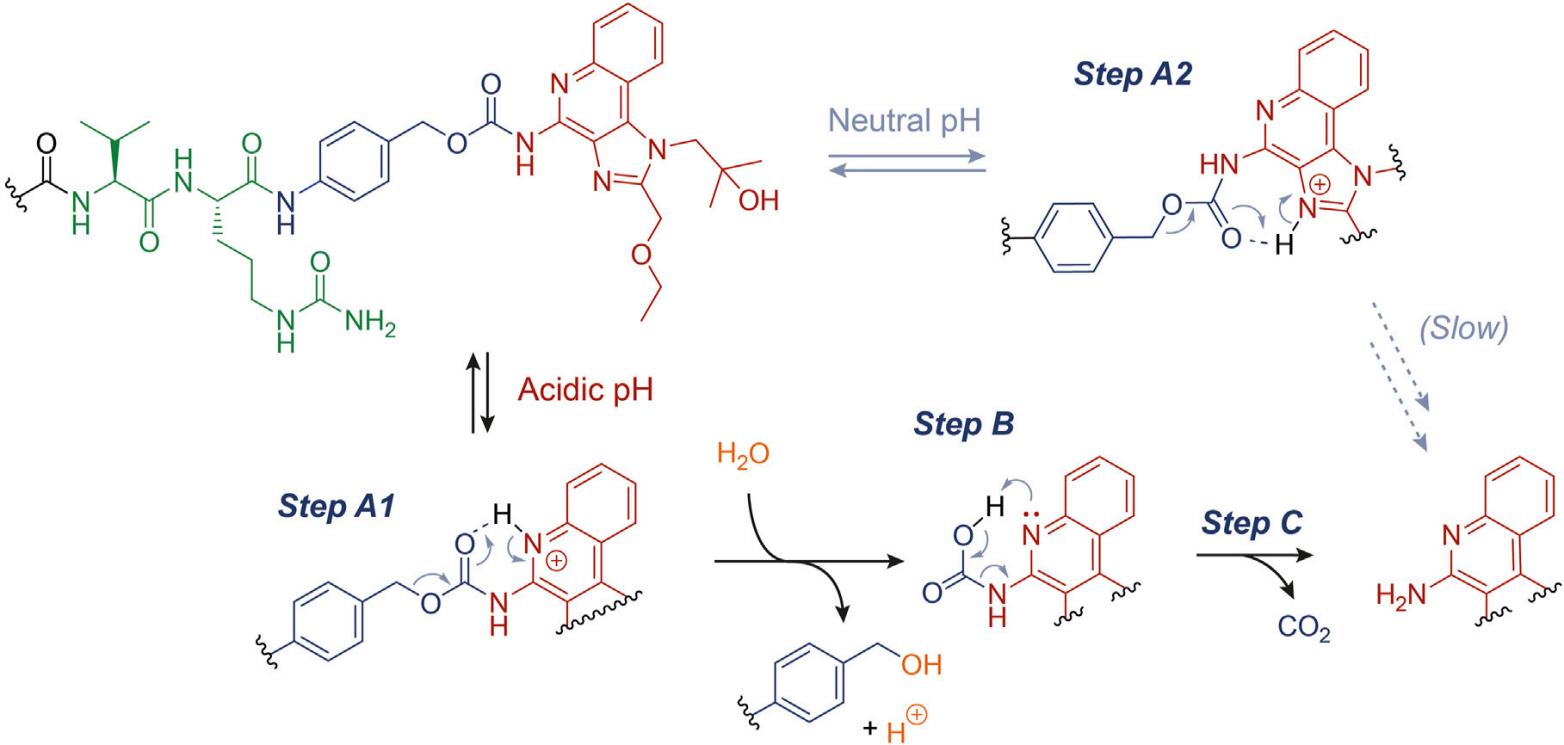
Proposed mechanism for the acid-catalyzed hydrolysis of the PABC-R848 carbamate bond.

We propose a plausible rationale for this observation, as described in Figure 3. In particular, the acidic environment conceivably favors the protonation (Step A1) of the quinoline N atom (p*K*_a_ ∼ 4), which may assist the cleavage of the C-O bond forming a 6-membered ring and the release of the benzyl carbocation, which would be ultimately quenched by water (Step B) (Ramon et al., 2014). At this stage, the carbamate decarboxylation (Step C) would result in the free R848. We speculate that this drug release mechanism, alternative to an enzymatic digestion, may enhance the R848 concentrations in the tumor microenvironment. Indeed, not only have acid-labile connections been extensively proposed for a number of drug delivery technologies, but they can also represent an asset whenever the enzymatic linker cleavage does not proceed quantitatively *in vivo* (Dal Corso et al., 2019; Migliorini et al., 2022). However, the marginal R848 release under neutral pH (Figure 1D) suggests that the carbamate cleavage also takes place when the quinoline N atom is not significantly protonated. In this case, following a similar degradation mechanism to the one described above, the carbamate cleavage may be assisted by the protonated imidazole ring (p*K*_a_ ∼ 7). The resulting rearrangement (Step A2) would proceed through the formation of an unfavored 7-membered ring, explaining the higher carbamate stability under neutral conditions than at lower pH values.

Finally, our *ex vivo* biodistribution analysis confirmed the structural weakness of the ISAC **3**. More specifically, the very low amount of free R848 detected in both tumor and plasma (Figure 2D) was in stark contrast to the high %ID/g of total IgG measured in the same organs (Figure 2E). The amount of free R848 detected in excretory organs (i.e., kidney and liver) decreased from 6 to 24 h post-injection, but the low %ID/g values at this timepoint indicated that a rapid release of R848 from ISAC **3** occurred in plasma at earlier stages. Despite the significant and preferential tumor accumulation of the anti-CAIX mAb, a preliminary analysis of tumor volume growth indicated a negligible anticancer activity of ISAC **3** (data not shown), in agreement with the low amount of free payload detected in the tumor. No significant body weight loss was observed in the treated animals, which indicated that ISAC **3** was well tolerated.

## 5 Conclusion

The structural optimization of the three individual units mAb, linker, and payloads and their assembly in tumor-targeting conjugates (such as ADCs and ISACs) impact on the overall therapeutic efficacy and toxicity. Aiming at a straightforward analysis of the structure activity relationships of these conjugates, novel techniques are being continuously proposed to evaluate their *in vivo* performance at the preclinical level as well as the payload accumulation at the site of disease (Dal Corso et al., 2017; Cahuzac and Devel, 2020). The *ex vivo* biodistribution analysis presented in this article indicates that the low stability of the ValCit-PABC-R848 linker-payload module may not be suited for the development of IgG-based ISAC products. However, the same linker-drug combination may be considered for other classes of active-targeting carriers characterized by a rapid tumor accumulation and low circulatory half-lives, such as tumor-targeting small molecules/peptides or small-format mAbs.

## 6 Scope statement

Immune-stimulating antibody conjugates (ISACs) represent an innovative class of biotherapeutics, similar to the more popular antibody drug conjugates (ADCs), but in this case immunostimulatory small molecules are used as payloads, rather than traditional cytotoxic agents. These novel payloads induce anti-tumor immune responses in the tumor environment, but they may also cause an excessive and uncontrolled activation of immune cells at the systemic level. In this work, we describe a novel ISAC featuring the protease-cleavable linker Valine-Citrulline (Val-Cit) connected to the aniline group of Resiquimod (R848) which is the key pharmacophoric portion of the molecule through a *para*-aminobenzyl carbamate (PABC) bond. Interestingly, this prodrug design led to the cargo release not only in the presence of the protease Cathepsin B, but also under acidic conditions. To the best of our knowledge, this low stability of the PABC-R848 connection is described for the first time and it likely results from the unique structural features of imidazoquinoline family of compounds. The quantitative biodistribution analysis of the ISAC in tumor-bearing mice was performed *ex vivo*, using a mass spectrometry protocol. Our data confirmed the low stability of the linker-drug connection while highlighting the long circulatory half-life of the antibody and its slow and selective accumulation in the solid tumor. This data provides important structural information for the design of new-generation ISACs.

## Data Availability

The datasets presented in this study can be found in online repositories. The names of the repository/repositories and accession number(s) can be found in the article/Supplementary Material.
