# Supplementary material for: Ex vivo mass spectrometry-based biodistribution analysis of an antibody-Resiquimod conjugate bearing a protease-cleavable and acid-labile linker
Source: Front Pharmacol. 2023 Dec 6;14:1320524. doi: 10.3389/fphar.2023.1320524 (PMC10731371; doi:10.3389/fphar.2023.1320524)

Supplementary Material

Ex Vivo Mass Spectrometry-Based Biodistribution Analysis of an Antibody-Resiquimod Conjugate Bearing a Protease-Cleavable and Acid-Labile Linker

Lydia Bisbal Lopez,[1] Domenico Ravazza,[2] Matilde Bocci,[2] Aureliano Zana,[2] Lucrezia Principi,[2] Sheila Dakhel Plaza,[2] Andrea Galbiati,[2] Ettore Gilardoni,[2] Jörg Scheuermann,[3] Dario Neri,[2,3,4] Luca Pignataro,[1] Cesare Gennari,[1] Samuele Cazzamalli[2*] and Alberto Dal Corso[1*]

[1]Chemistry Department, Università degli Studi di Milano, Milano, Italy

[2]R&D Department, Philochem AG, Otelfingen, Switzerland

[3]Department of Chemistry and Applied Biosciences, Swiss Federal Institute of Technology (ETH Zürich), Zürich, Switzerland

[4]Philogen S.p.A, Siena, Italy

*** Correspondence:**
Alberto Dal Corso / Samuele Cazzamalli
alberto.dalcorso@unimi.it / samuele.cazzamalli@philochem.ch

Table of Contents

1. List of Abbreviations and Symbols 2

2. General Remarks and Procedures 3

3. ISAC **3** Characterization 3

4. Appendix 5

The datasets generated for this study can be found in the Dataverse repository of the Università Degli Studi di Milano at this link.

1. List of Abbreviations and Symbols

Cit	Citrulline
CV	Column Volume
ESI	Electrospray Ionization
HPLC	High Performance Liquid Chromatography
HR	High Resolution
h	Hours
LC-MS	Liquid Chromatography - Mass Spectrometry
LR	Low Resolution
m/z	Mass-To-Charge Ratio
Mc	Maleimidocaproyl
MS	Mass Spectroscopy
mQ	Milli-Q
PABC	*para*-Aminobenzoyl Carbamate
PBS	Phosphate-Buffered Saline
PNP	*para*-nitrophenyl carbonate
R848	Resiquimod
SDS	Sodium Dodecyl Sulfate
SDS-PAGE	Sodium Dodecyl Sulfate – Polyacrylamide Gel Electrophoresis
t	Time
Val	L-Valine

2. General Remarks and Procedures

Mc-ValCit-PABC-PNP was purchased from MedChemExpress, Resiquimod (R848) was purchased from Fluorochem and Cathepsin B (from human placenta) was purchased from Merck. All other reagents were purchased from Merck and used as supplied. Solvents were used as supplied by Merck in HPLC or analytical grade.

HPLC purifications were performed on Dionex Ultimate 3000 equipped with Dionex RS Variable Wavelength Detector (column: Atlantis Prep T3 OBDTM 5 µm 19 x 100 mm; flow 10 mL/min; solvent A: H_2O + 0.1% AcOH, solvent B: MeCN). Stability analyses were performed on a Waters 515 HPLC pumps equipped with a 996 photodiode array detector and a Waters Atlantis T3 - 5 µm - 4.6 x 100 mm column (injection volume: 150 µL; UV analysis: 254 nm).

High Resolution mass spectrometry analyses (4 decimal places) were performed at the Mass Spectrometry facility of the Unitech COSPECT at the University of Milan (Italy) on a SYNAPT G2-Si QToF instrument (equipped with a ZsprayTM ESI-probe) (Waters, Milford, MA, USA) coupled with an Acquity UPLC I-Class chromatography system (Waters) and an Acquity UPLC PDA Detector (Waters). Low resolution mass spectra (1 and 2 decimal places) were recorded on a Thermo Scientific LCQ Fleet Ion Trap Mass Spectrometer (ESI source).

3. ISAC Characterization

3.1. SDS-PAGE

Protein samples were diluted to 0.2-0.3 mg/ml in PBS and mixed with either Reducing or Non-Reducing 5x Loading buffer. Samples were denatured for 5 min at 95°C and loaded on NuPAGE 4-12% Bis-Tris Gel (NovexTM by Life Technologies). 1x MES NuPAGE (NovexTM by Life Technologies) was used as running buffer. The electrophoresis was performed at 180 V, 110 mA for 1 h. The gel was then rinsed with deionized water and stained with Coomassie blue for 15 min on an orbital shaker. The staining solution was discarded. The gel was then rinsed with deionized water and immerged in destaining solution (10% AcOH / 30% MeOH / mQ water) for 3 h on an orbital shaker. The destaining solution was discarded and recycled and the gel was rinsed with deionized water. Recipes for loading buffers and staining solution are described in Tables S1-S3.

5x Non-Reducing Loading Buffer (100 mL)

Tris·HCl (250 mM, pH 6.8)	20.8 mL
Glycerol	33.3 mL
SDS	6.6 mg
Bromophenol Blue	66 mg
mQ H_2O	Up to 100 mL

Table S1. 5x Non-Reducing Loading buffer recipe

**5x Reducing Loading Buffer (100 mL)**	
Tris·HCl (250 mM, pH 6.8)	20.8 mL
Glycerol	33.3 mL
SDS	6.6 mg
Bromophenol Blue	66 mg
mQ H_2O	Up to 100 mL
β-mercaptoethanol	10% (*v/v*)

Table S2. 5x Reducing Loading buffer recipe

**Coomassie Blue Staining (1 L)**	
PlusOne Coomassie PhastGel Blue R-350	2 tablets
MeOH	400 mL
AcOH	100 mL
mQ H_2O	500 mL

Table S3. Coomassie blue staining recipe

3.2. Gel Filtration

A 100 µL of diluted ISAC **3** sample (final concentration 0.1-0.5 mg/mL) was loaded on FPLC (Äkta, GE Healthcare) and the protein was separated by a Superdex200 Increase 10/300 GL column (GE Healthcare) previously equilibrated with 1 CV PBS, using PBS as the mobile phase at a flow rate of 0.6 mL/min (column pressure limit set at 5 MPa). The protein was detected by a UV-detector at a wavelength of 280 nm.

3.3. Mass Spectrometry

A sample of ISAC **3** was diluted to about 0.1 mg/mL and LC-MS was performed on a Waters Xevo G2XS QTof instrument (ESI-ToF-MS) coupled to a Waters Acquity UPLC H-Class System using a 2.1 × 50 mm Acquity BEH300 C4 1.7 µm column (Waters). H_2O + 0.1% FA (solvent A) and MeCN + 0.1% FA (solvent B) were used as the mobile phase at a flow rate of 0.4 mL/min. The gradient was programmed as follows: after 1.5 min isocratic with 95% solvent A, stepwise change from 95% solvent A to 95% solvent B in 4.5 min (10% increase every 0.5 min), back to 95% solvent A in 0.5 min, linearly to 95% solvent B and back to 95% solvent A in 2.25 min (last step repeated twice).

4. Appendix

4.1. HPLC Purity Analysis

4.1.1. Mc-ValCit-PABC-R848 ***1***

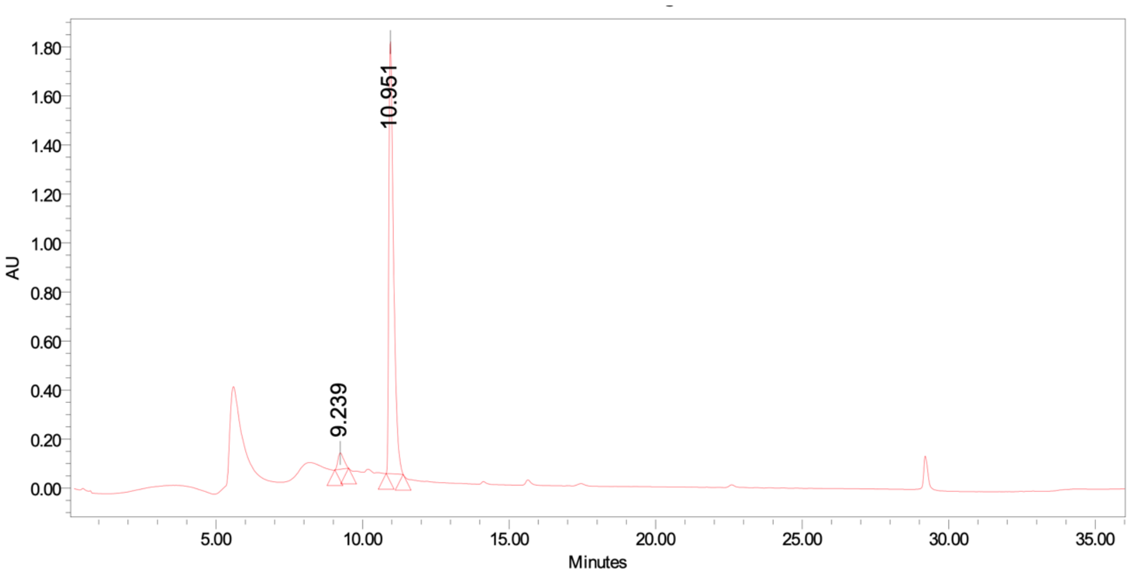

Compound	t_R (min)	Area	Height	% Area
R848	9.239	910496	66646	4.42
Mc-ValCit-PABC-R848 **1**	10.951	19706280	1765069	95.58

4.1.2. Cys-ValCit-PABC-R848 ***2***

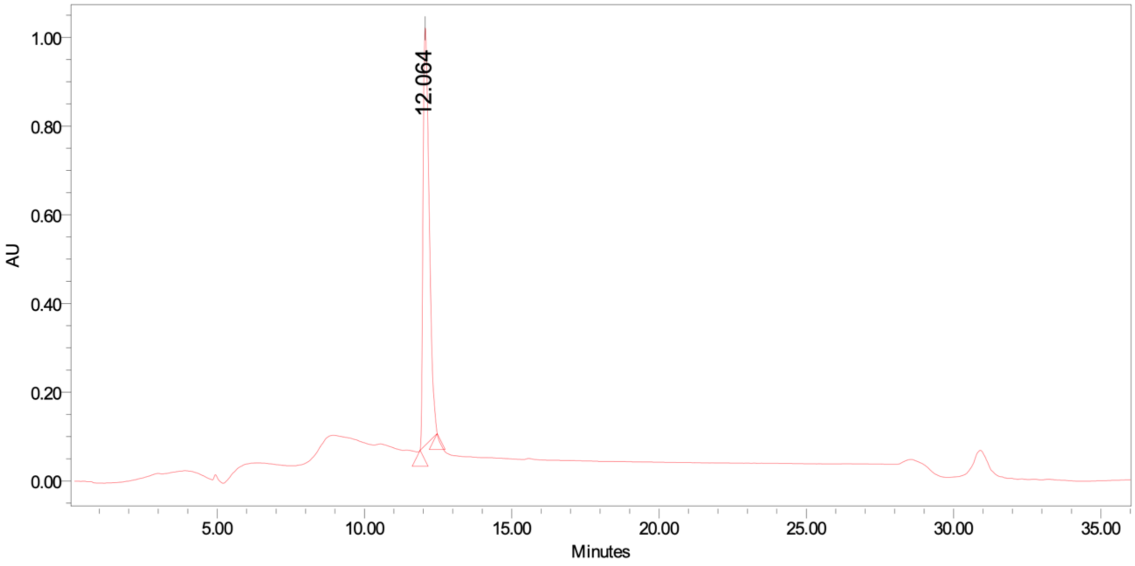

Compound	t_R (min)	Area	Height	% Area
Cys-ValCit-PABC-R848 **2**	12.064	13654987	946631	100

4.2. Prodrug Stability Data Overview

4.2.1. HPLC Analysis

The samples were injected in into an analytical HPLC-PDA system (see General Remarks and Procedures). H_2O + 0.1% TFA (solvent A) and MeCN + 0.1% TFA (solvent B) were used as the mobile phase at a flow rate of 1 mL/min. The gradient was programmed from 5% to 30% B over 26 min.

Areas under the curve (AUC) of the detected peaks were measured using software associated to the HPLC systems. The rate of R848 release from the starting carbamate was obtained by calculating the relative ratios of AUC values corresponding to the prodrug **2** and the free payload. Data were plotted versus time using GraphPad Prism software.

4.2.2. Cathepsin B Cleavage Assay of ***2***

**Timepoint**	**% Area 2**
t = 0	99.14
t = 2 h	43.37
t = 4 h	3.47
t = 5 h	1.37

4.2.3. Stability of ***2*** *in Acetate Buffer (pH 5.4)*

**Timepoint**	**% Area 2**
t = 0	97.86
t = 4 h	92.51
t = 24 h	68.13

4.2.4. Stability of ***2*** *in Acetate Buffer (pH 3.8)*

**Timepoint**	**% Area 2**
t = 0	98.81
t = 4 h	58.01
t = 24 h	1.75

4.2.5. Stability of ***2*** *in Phosphate Buffer (pH 7.4)*

**Timepoint**	**% Area 2**
t = 0	99.01
t = 4 h	97.16
t = 24 h	84.84

4.3. HR LC-MS Analysis

4.3.1. UPLC-PDA-ESI-HR-MS Analysis

Samples at timepoints 0 and 4 h for the Cathepsin B release assay and stability in Acetate Buffer (2.2 M, pH 5.4) were submitted for High Resolution Mass Spectrometry (see General Remarks and Procedures).

Chromatographic separation was carried out on an ACQUITY UPLC HSS T3 column (100 x 2.1 mm, 1.8 µm, 30 °C) (Waters) fitted with a VanGuard cartridge (Waters) with a gradient program from 100% A (H_2O + 0.1% FA), 0% B (MeCN + 0.1% FA) to 30% A, 70% B in 13.5 minutes. Ionization was carried out on an ESI positive mode with the following conditions: capillary 3 kV, sampling cone 80, source temperature 120 °C, desolvatation temperature 150 °C, desolvatation gas flow 600 L/h. PDA (wavelength range: 190–410 nm) measurements were taken at 254 nm. The mass spectrometer operated with the following parameters: analyser mode High Resolution, scan range 50–1500 m/z, lock mass compound leucine enkephalin. Samples (2 µL) were injected. Data was elaborated with MassLynxTM v4.2 software (Waters).

4.3.2. Pure Cys-ValCit-PABC-R848 ***2***

LC profile (254 nm)

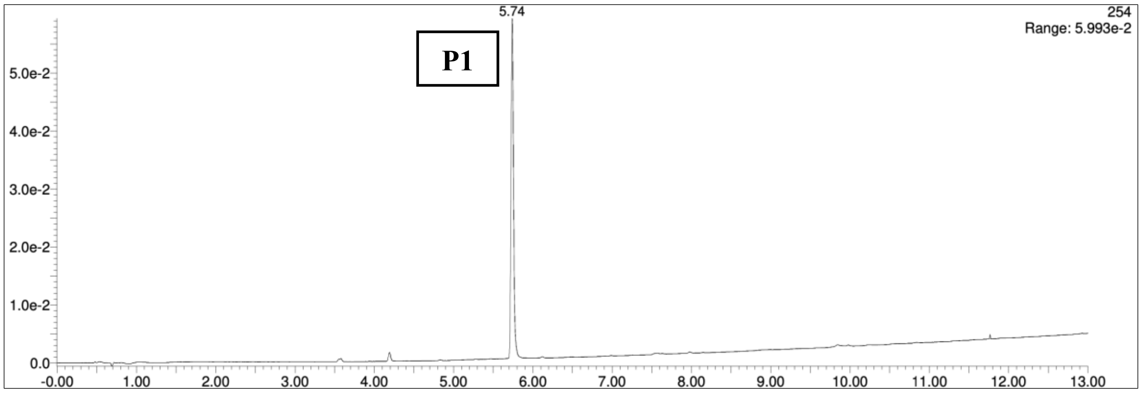

P1 (t_R = 5.74 min) - MS Spectrum

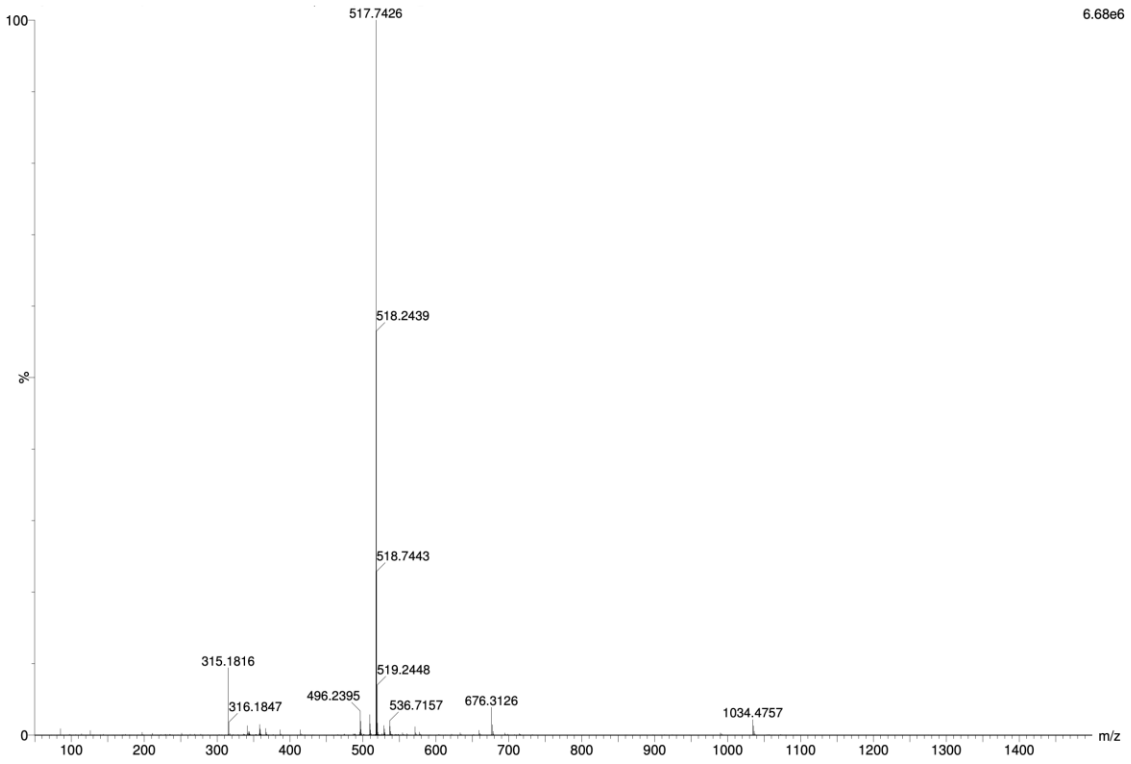

*4.3.3 Stability of **2** in Acetate Buffer (pH 5.4) + Cathepsin B (4 h incubation)*

LC profile (254 nm)

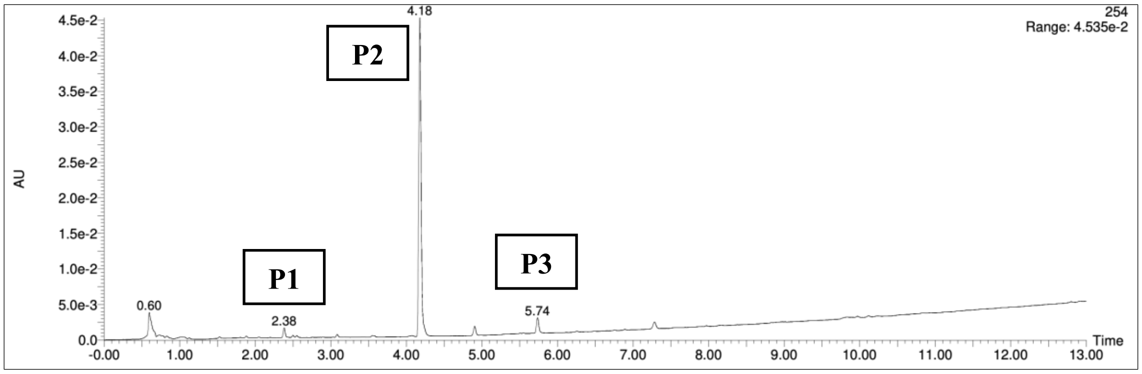

P1 (t_R = 2.38 min) - MS Spectrum

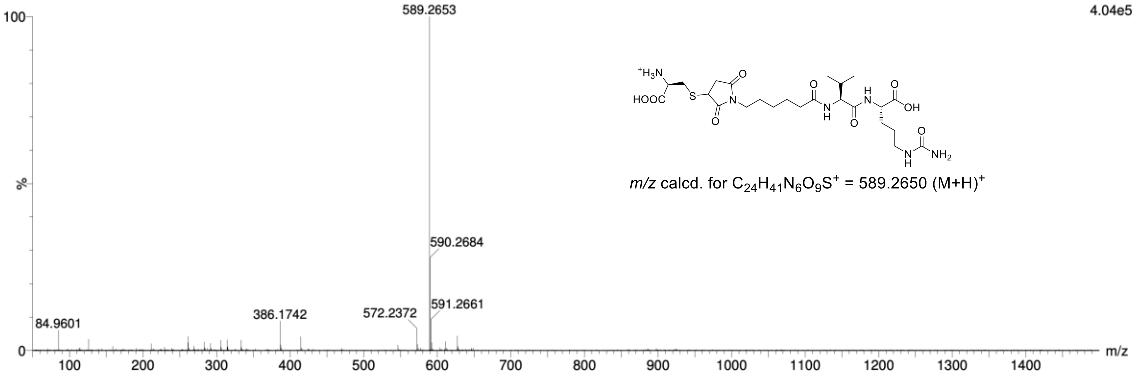

P2 (t_R = 4.18 min) - MS Spectrum

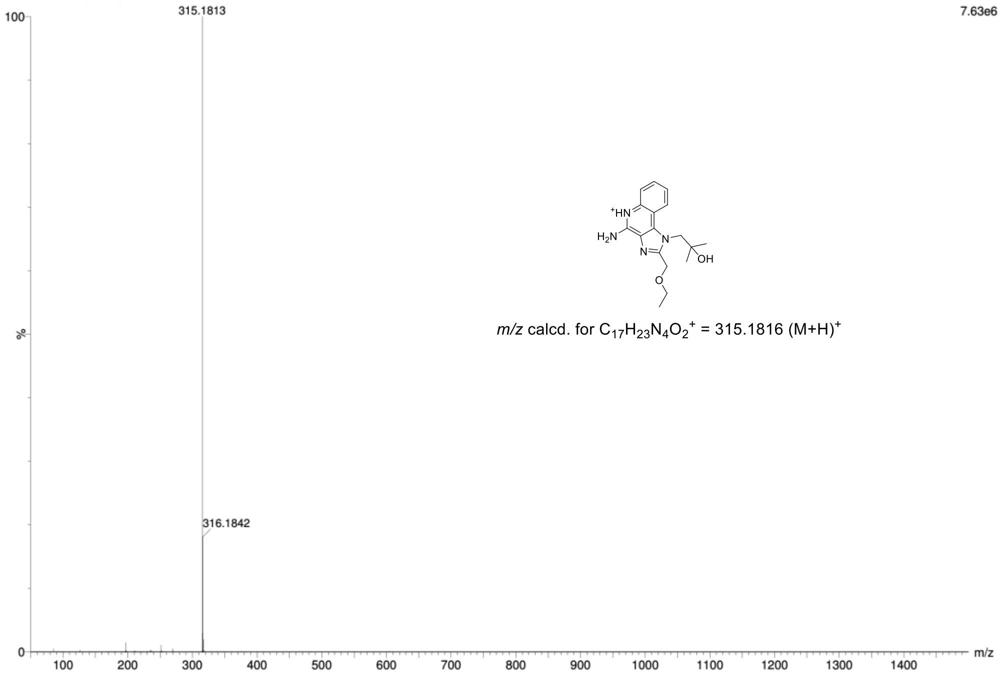

P3 (t_R = 5.74 min) - MS Spectrum

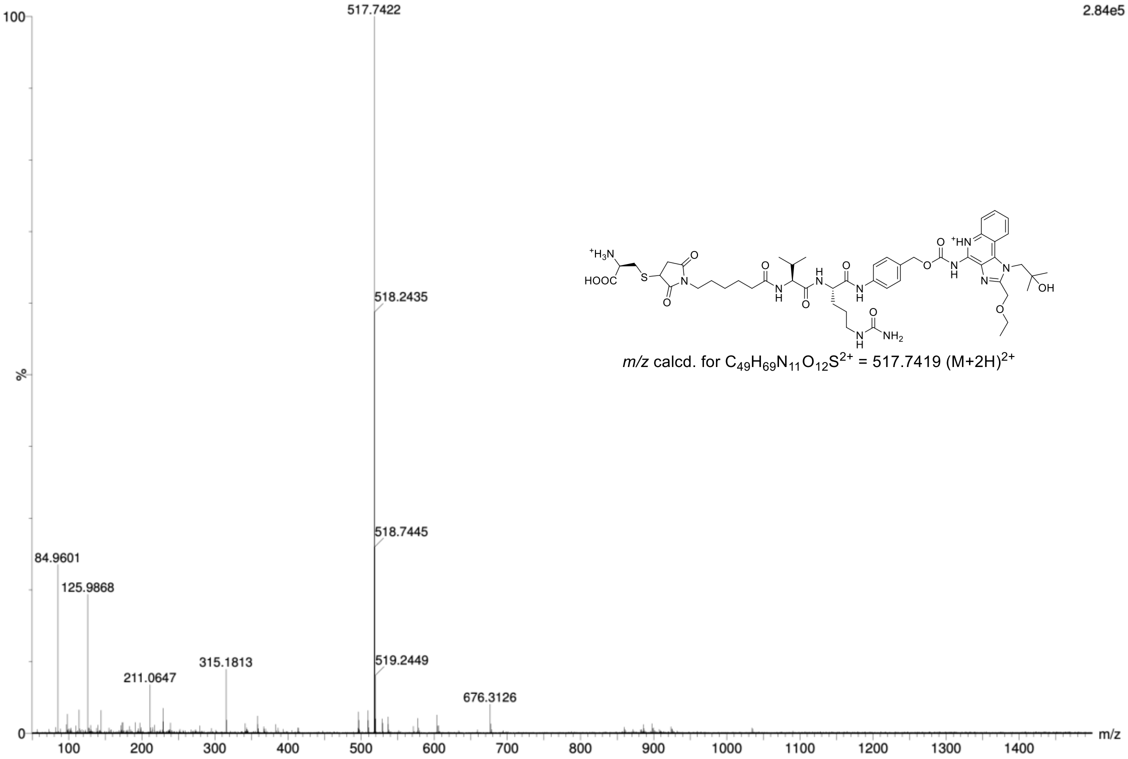

*4.3.4 Stability of **2** in Acetate Buffer (pH 5.4) (4 h incubation)*

LC profile (254 nm)

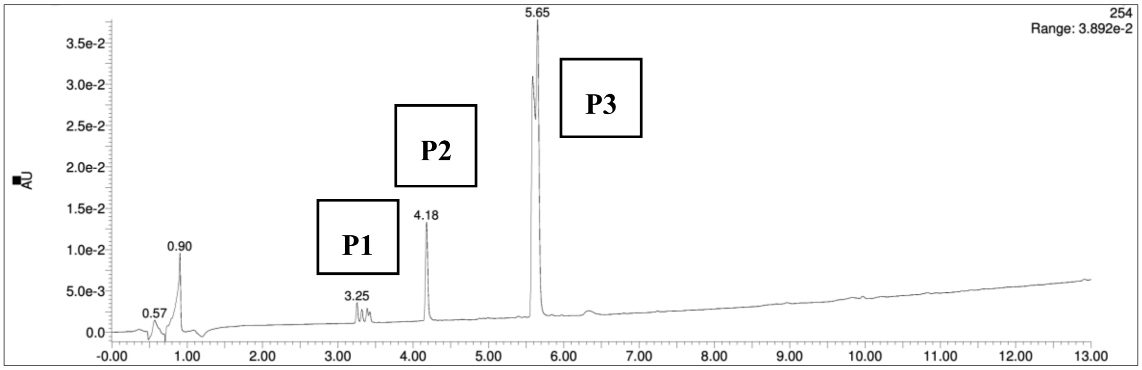

P1 (t_R = 3.25 min) - MS Spectrum

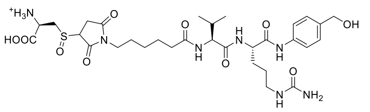

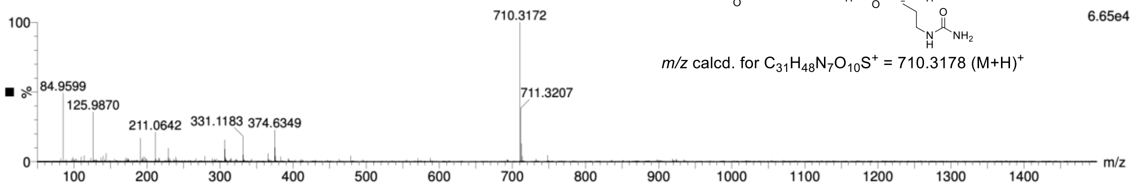

P2 (t_R = 4.18 min) - MS Spectrum

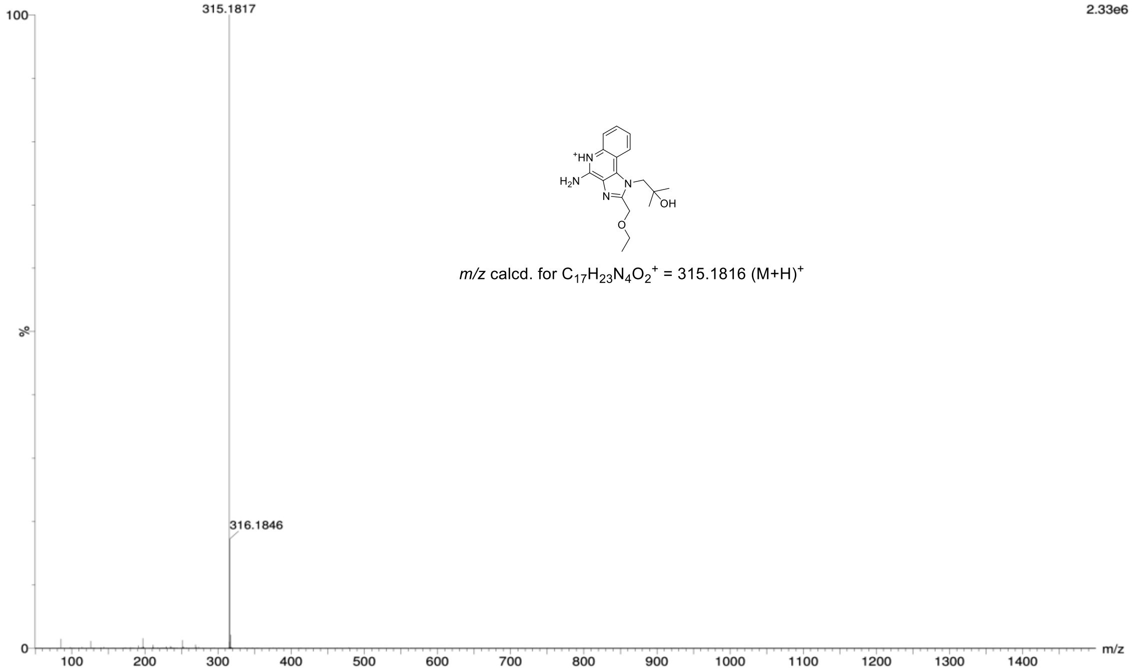

P3 (t_R = 5.65 min) - MS Spectrum

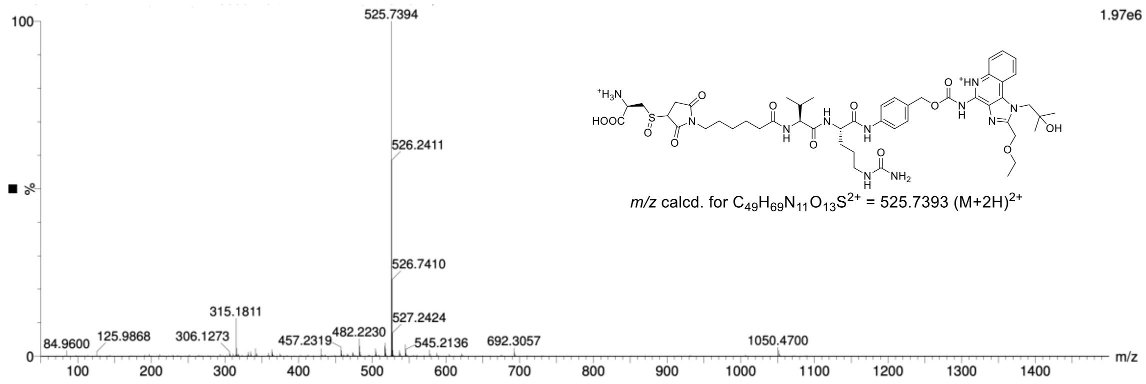

4.4. LR MS Analysis

4.4.1. Mc-ValCit-PABC-R848 ***1***

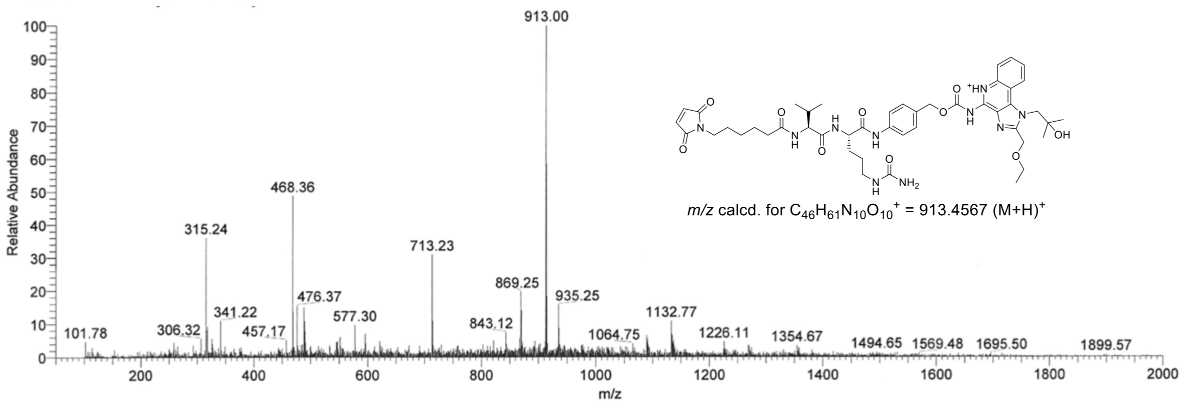

4.5. ISAC **3** Characterization

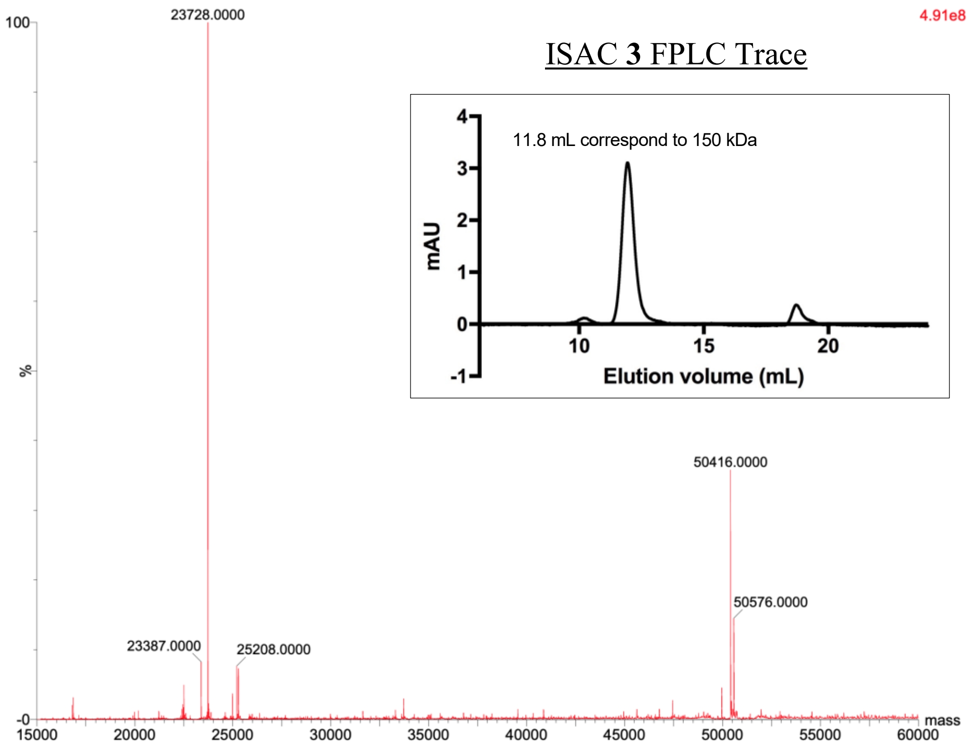

Supplement: Supplementary file 1 [file DataSheet1.pdf]
